# Supplementary material for: New Sorbicillinoids from the Mangrove Endophytic Fungus Trichoderma reesei SCNU-F0042
Source: Mar Drugs. 2023 Aug 5;21(8):442. doi: 10.3390/md21080442 (PMC10455806; doi:10.3390/md21080442)
Supplement: Supplementary file 1 [file marinedrugs-21-00442-s001.zip › marinedrugs-2534049-supplementary.pdf]

Supplementary data

New Sorbicillinoids from the Mangrove Endophytic Fungus *Trichoderma reesei* SCNU-F0042.

Jialin Li[1], Tao Chen[1], Jianchen Yu[2], Hao Jia[1], Chen Chen[1], Yuhua Long [1,*]

1 GDMPA Key Laboratory for Process Control and Quality Evaluation of Chiral Pharmaceuticals, School of Chemistry, South China Normal University, Guangzhou 510006, China; jialinli@m.scnu.edu.cn (J.L.); chent296@mail2.sysu.edu.cn (T.C.); haojia@m.scnu.edu.cn (H.J.); chenchen2021@m.scnu.edu.cn (C.C.);

2 Key Laboratory of Tropical Disease Control (Sun Yat-sen University), Ministry of Education, Guangzhou 510080, China; Department of Biochemistry, Zhongshan School of Medicine, Sun Yat-sen University, Guangzhou 510080, China; yujch3@mail.sysu.edu.cn (J.Y.)

* Correspondence: longyh@scnu.edu.cn

Contents

Figure.S1. ^{1}H NMR (methanol-d_4, 600 MHz) spectrum of compound **1** 1
Figure.S2. ^{13}C NMR (methanol-d_4, 150 MHz) spectrum of compound **1** 1
Figure.S3. ^{1}H, ^{1}H-COSY spectrum (methanol-d_4, 600 MHz) spectrum of compound **1** 2
Figure.S4. HSQC spectrum (methanol-d_4, 600 MHz) spectrum of compound **1** 2
Figure.S5. HMBC spectrum (methanol-d_4, 600 MHz) spectrum of compound **1** 3
Figure.S6. NOESY spectrum (methanol-d_4, 600 MHz) spectrum of compound **1** 3
Figure.S7. HR-ESI-MS spectrum of compound **1** 4
Figure.S8. ^{1}H NMR (methanol-d_4, 600 MHz) spectrum of compound **2** 4
Figure.S9. ^{13}C NMR (methanol-d_4, 150 MHz) spectrum of compound **2** 5
Figure.S10. ^{1}H, ^{1}H-COSY spectrum (methanol-d_4, 600 MHz) spectrum of compound **2** 5
Figure.S11. HSQC spectrum (methanol-d_4, 600 MHz) spectrum of compound **2** 6
Figure.S12. HMBC spectrum (methanol-d_4, 600 MHz) spectrum of compound **2** 6
Figure.S13. NOESY spectrum (methanol-d_4, 600 MHz) spectrum of compound **2** 7
Figure.S14. HR-ESI-MS spectrum of compound **2** 7
Figure.S15. ^{1}H NMR (methanol-d_4, 600 MHz) spectrum of compound **3** 8
Figure.S16. ^{13}C NMR (methanol-d_4, 150 MHz) spectrum of compound **3** 8
Figure.S17. ^{1}H, ^{1}H- COSY spectrum (methanol-d_4, 600 MHz) spectrum of compound **3** 9
Figure.S18. HSQC spectrum (methanol-d_4, 600 MHz) spectrum of compound **3** 9
Figure.S19. HMBC spectrum (methanol-d_4, 600 MHz) spectrum of compound **3** 10
Figure.S20. NOESY spectrum (methanol-d_4, 600 MHz) spectrum of compound **3** 10
Figure.S21. HR-ESI-MS spectrum of compound **3** 11
Figure.S22. ^{1}H NMR (acetone-d_6, 600 MHz) spectrum of compound **5**. 11
Figure.S23. ^{13}C NMR (acetone-d_6, 150 MHz) spectrum of compound **5** 12
Figure.S24. ^{1}H, ^{1}H- COSY spectrum (acetone-d_6, 600 MHz) spectrum of compound **5** 12
Figure.S25. HSQC spectrum (acetone-d_6, 600 MHz) spectrum of compound **5** 13
Figure.S26. HMBC spectrum (acetone-d_6, 600 MHz) spectrum of compound **5** 13
Figure.S27. HR-ESI-MS spectrum of compound **5** 14
Figure.S28. Molecular networking of the EtOAc extract from strain *Trichoderma reesei SCNU-F0042* 15

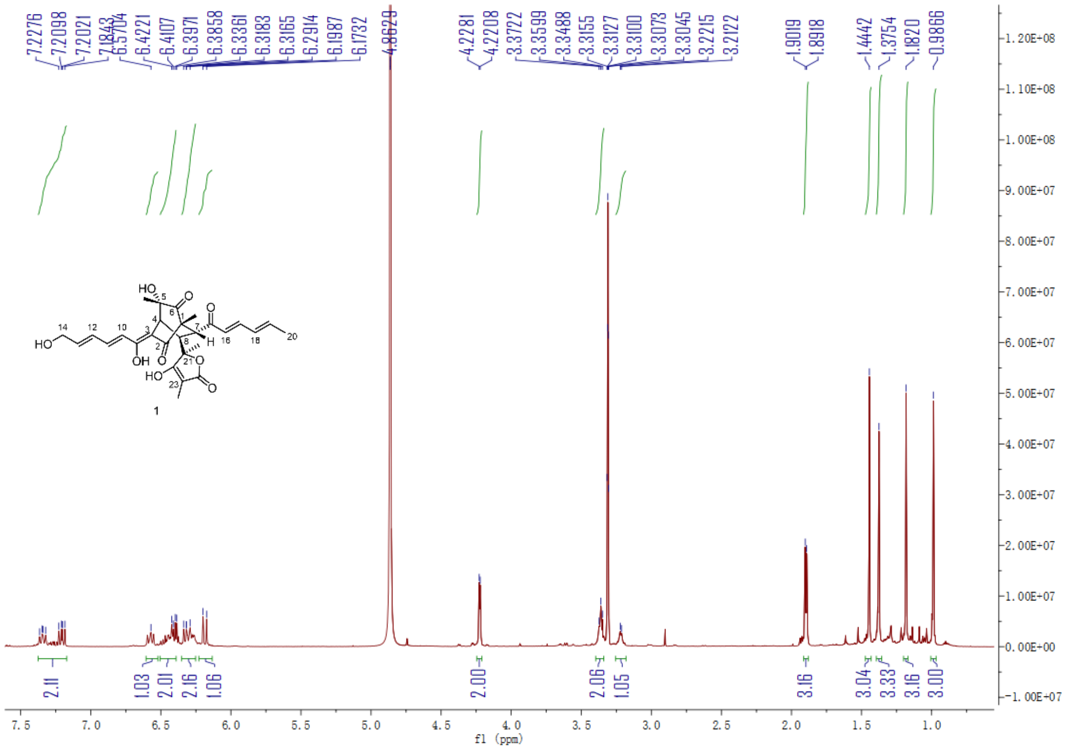

Figure.S1. ^{1}H NMR (methanol-d_4, 600 MHz) spectrum of compound **1**

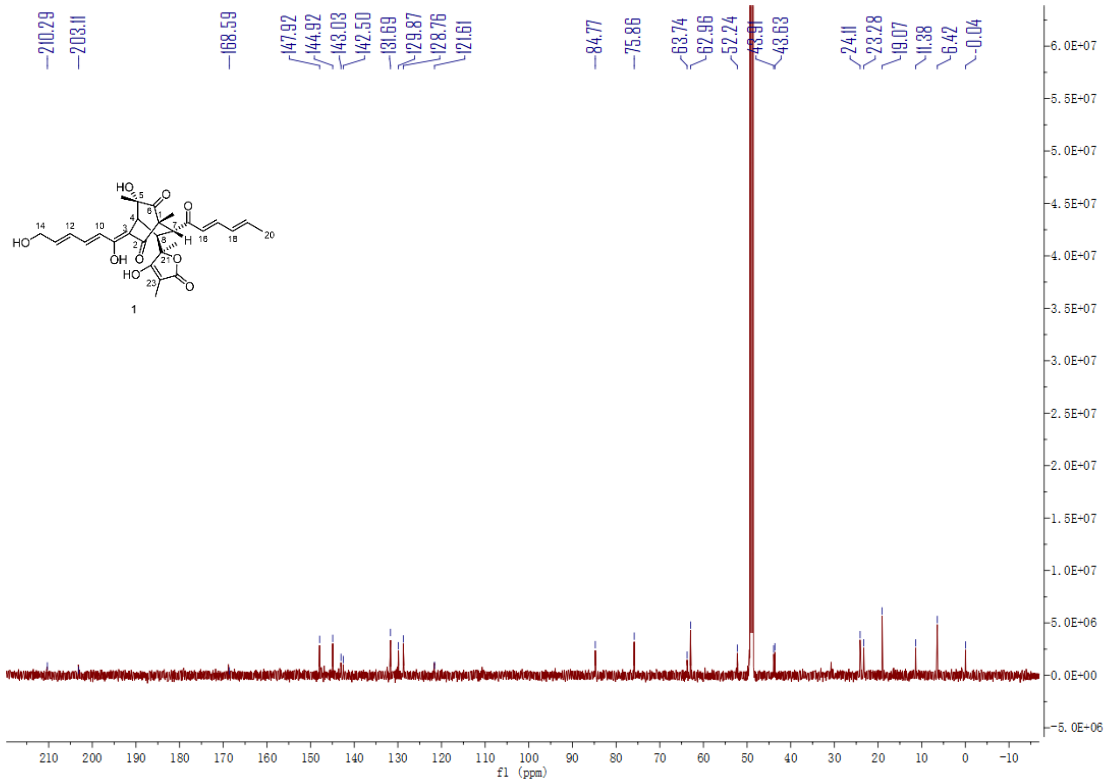

Figure.S2.^{13}C NMR (methanol-d_4, 150 MHz) spectrum of compound **1**

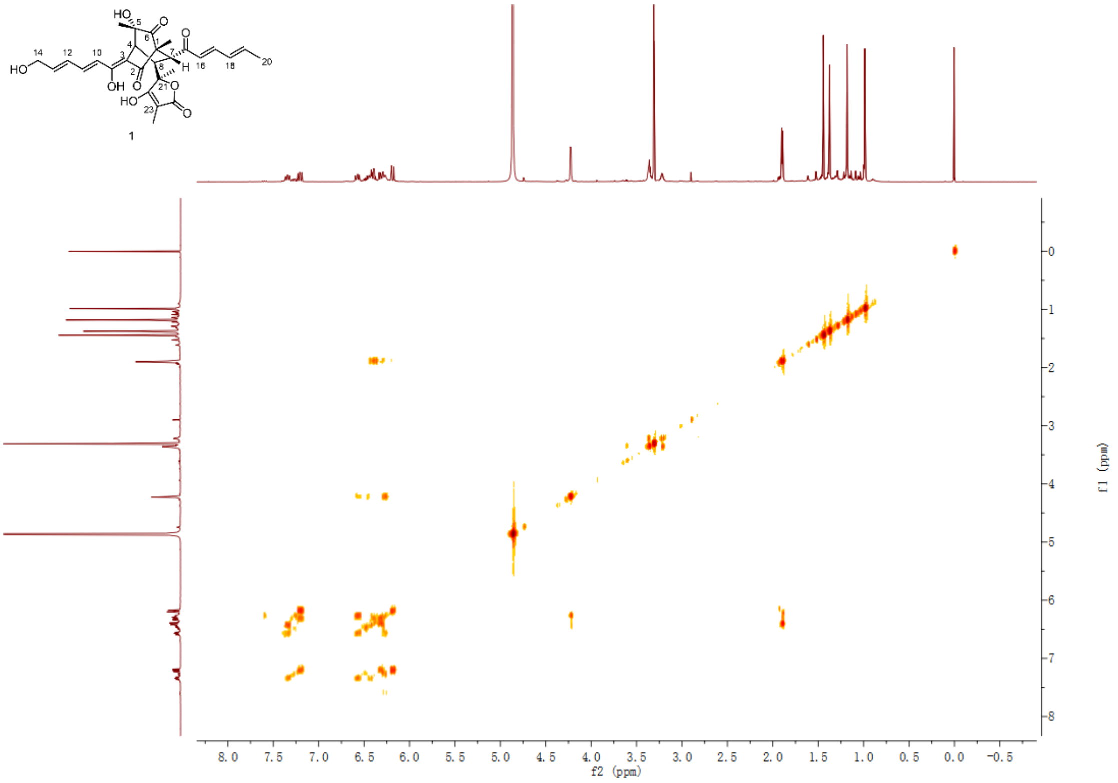

Figure.S3. ^{1}H, ^{1}H-COSY spectrum (methanol-d_4, 600 MHz) spectrum of compound **1**

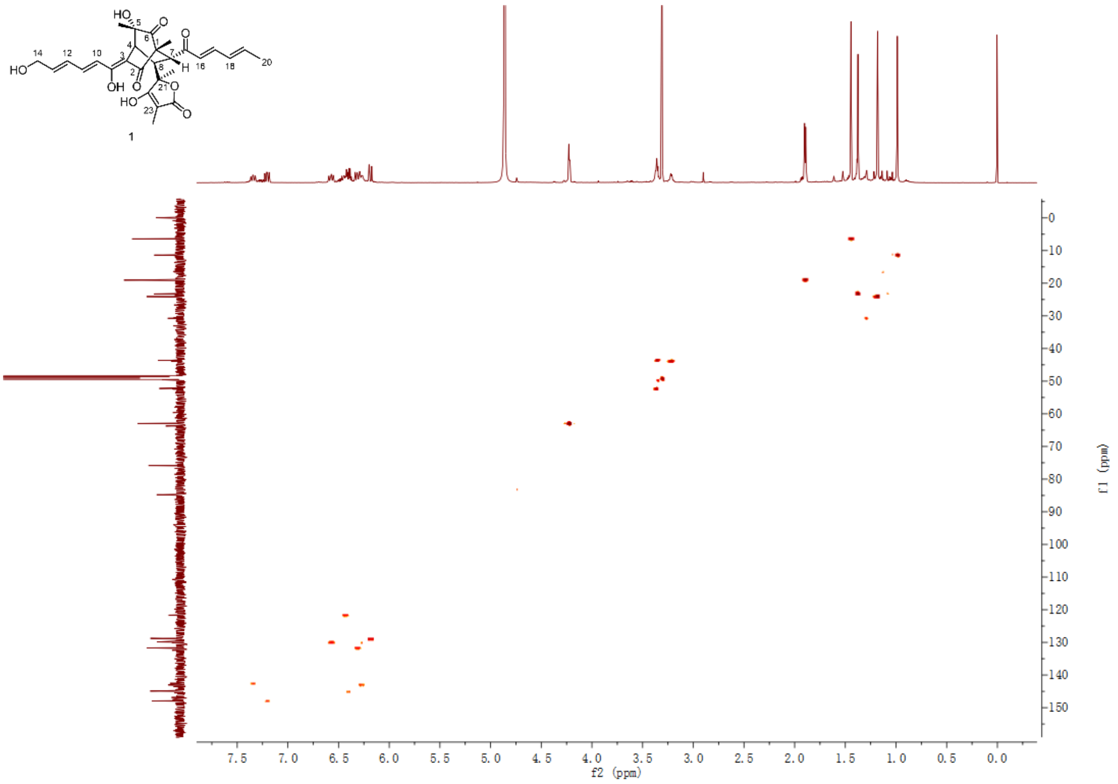

Figure.S4. HSQC spectrum (methanol-d_4, 600 MHz) spectrum of compound **1**

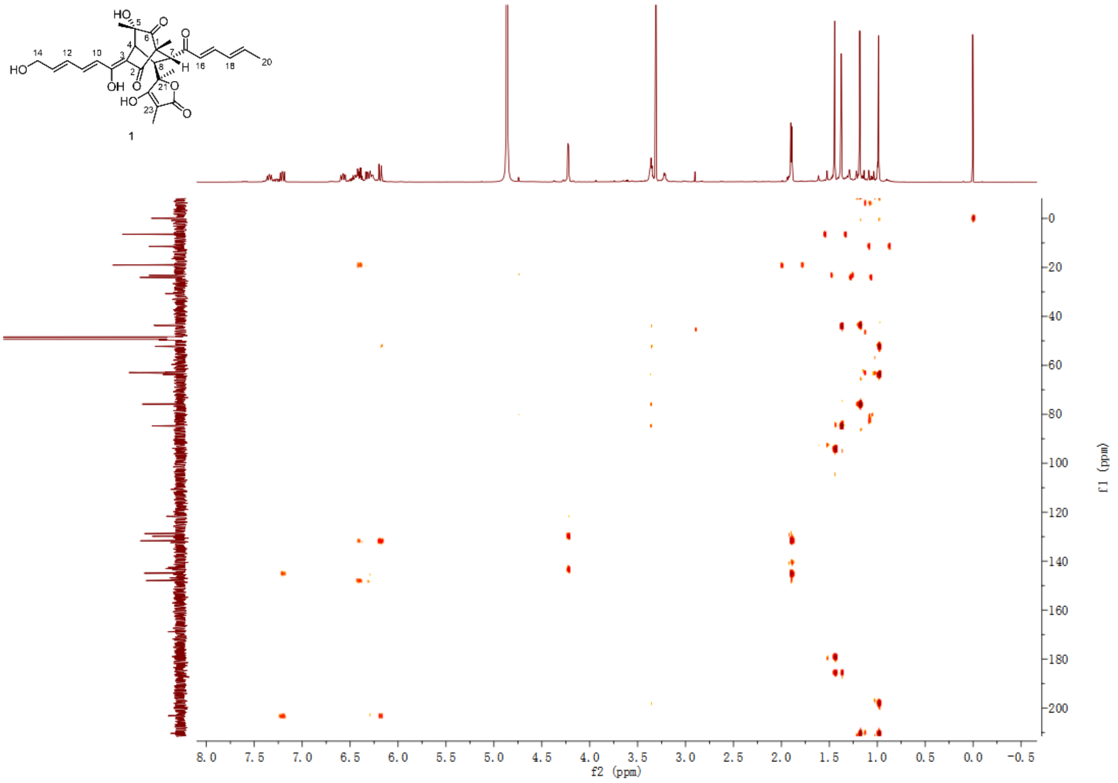

Figure.S5. HMBC spectrum (methanol-d_4, 600 MHz) spectrum of compound **1**

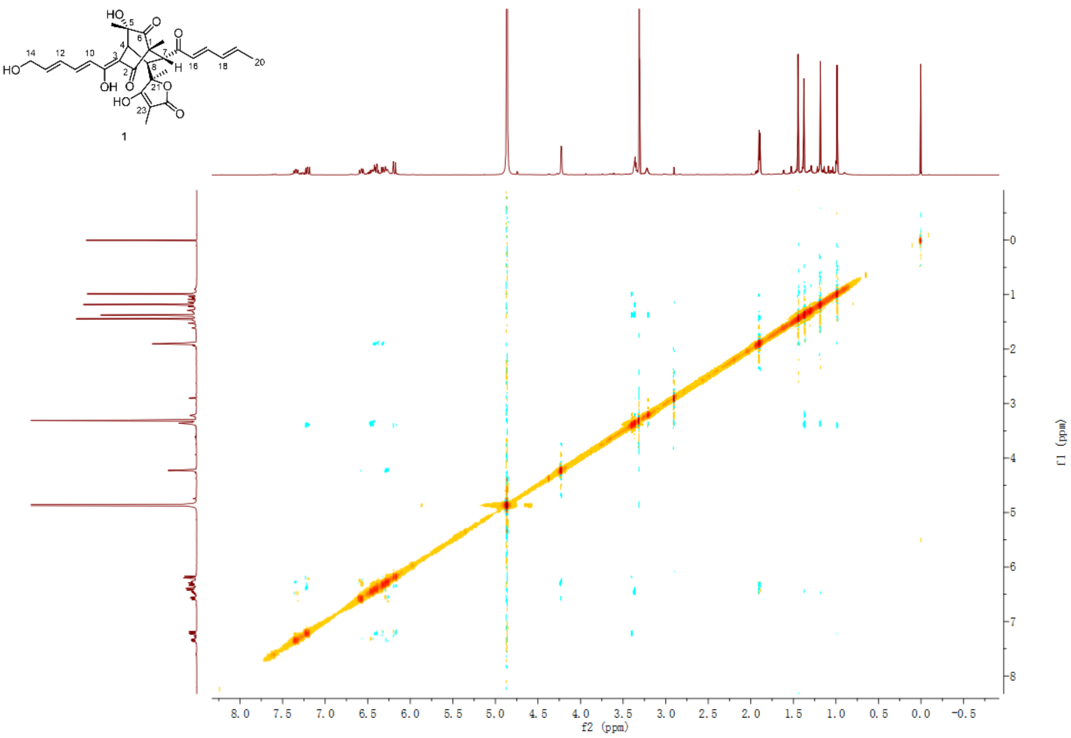

Figure.S6. NOESY spectrum (methanol-d_4, 600 MHz) spectrum of compound **1**

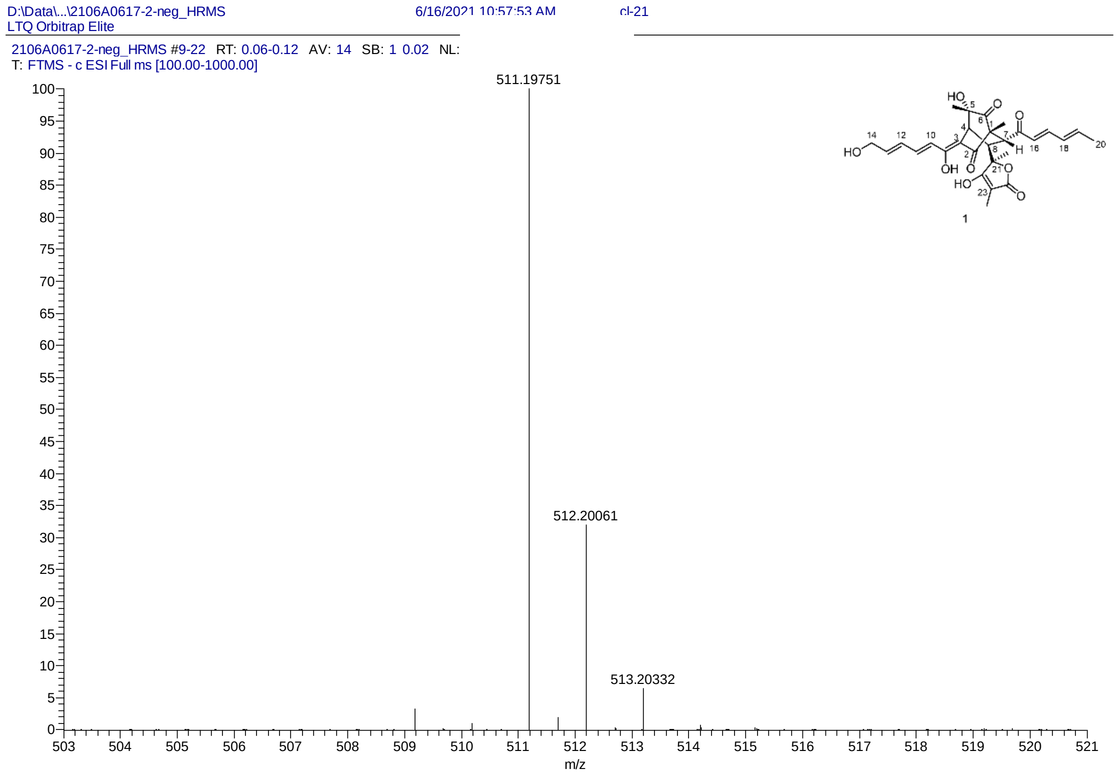

Figure.S7. HR-ESI-MS spectrum of compound **1**

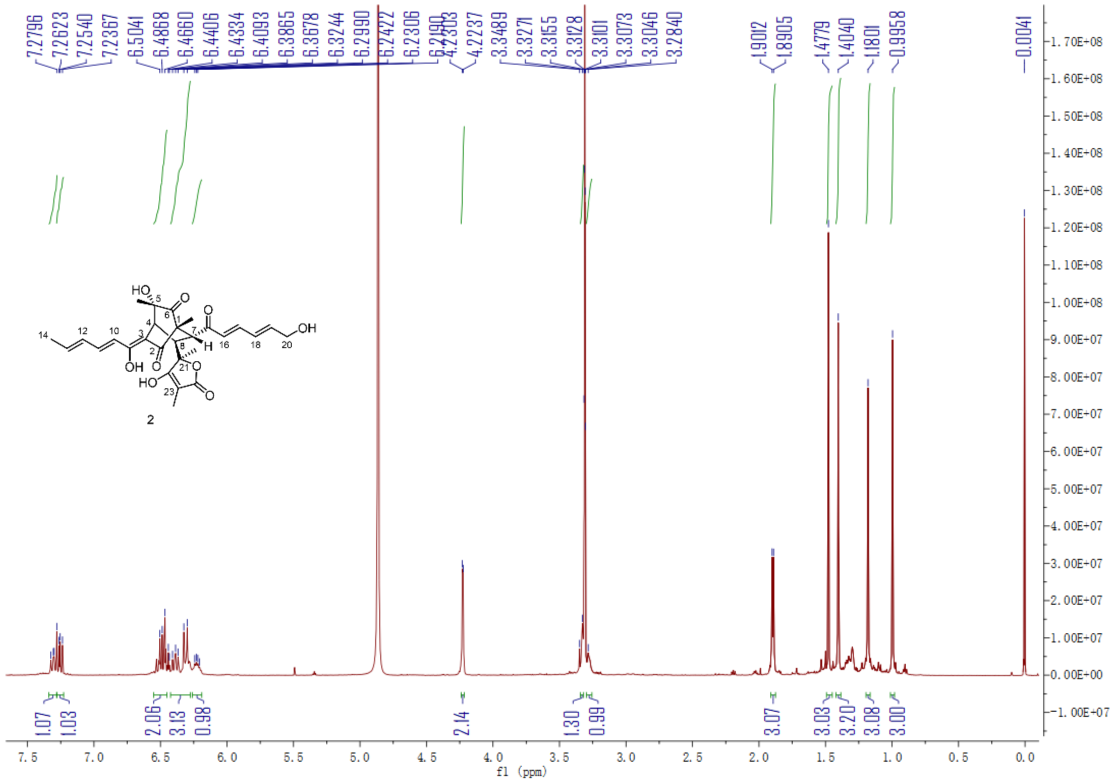

Figure.S8. ^{1}H NMR (methanol-d_4, 600 MHz) spectrum of compound **2**

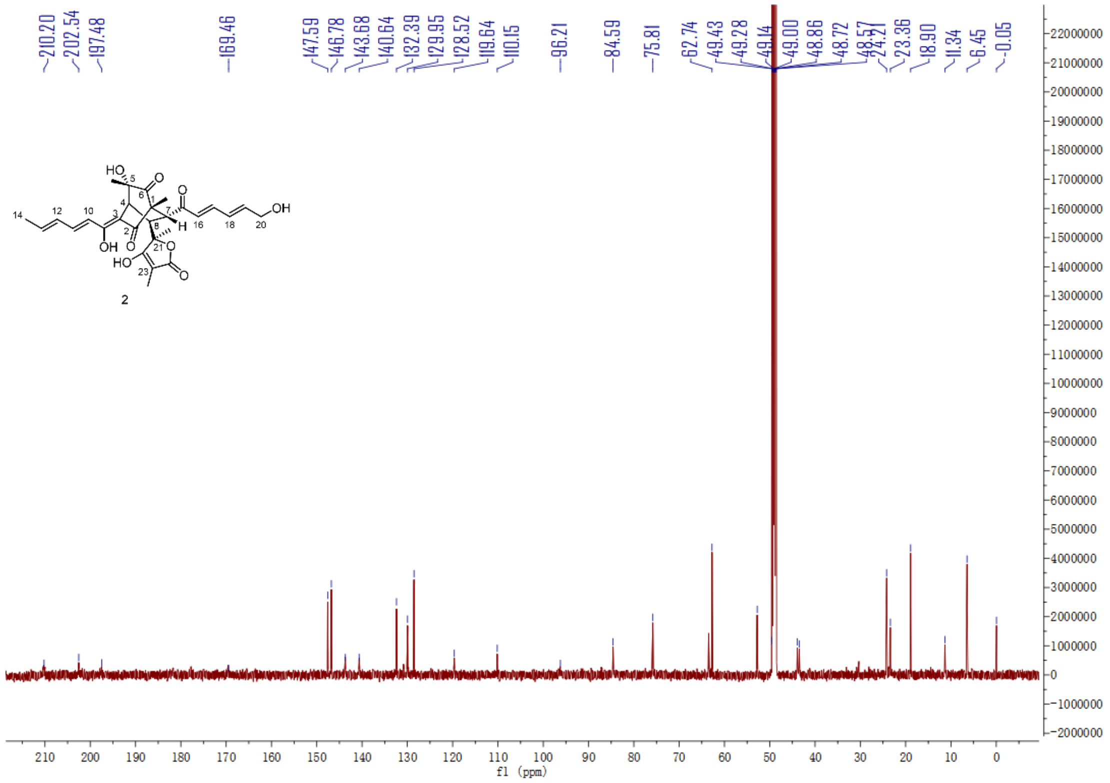

Figure.S9.^{13}C NMR (methanol-d_4, 150 MHz) spectrum of compound **2**

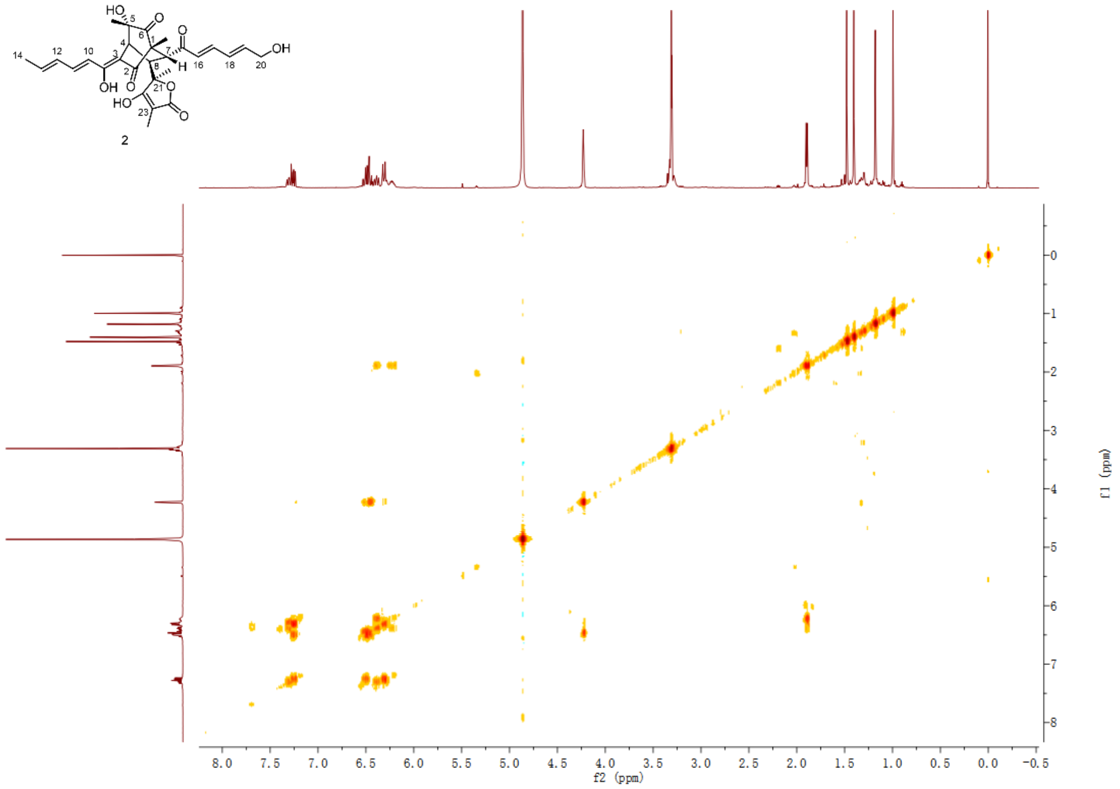

Figure.S10. ^{1}H, ^{1}H-COSY spectrum (methanol-d_4, 600 MHz) spectrum of compound **2**

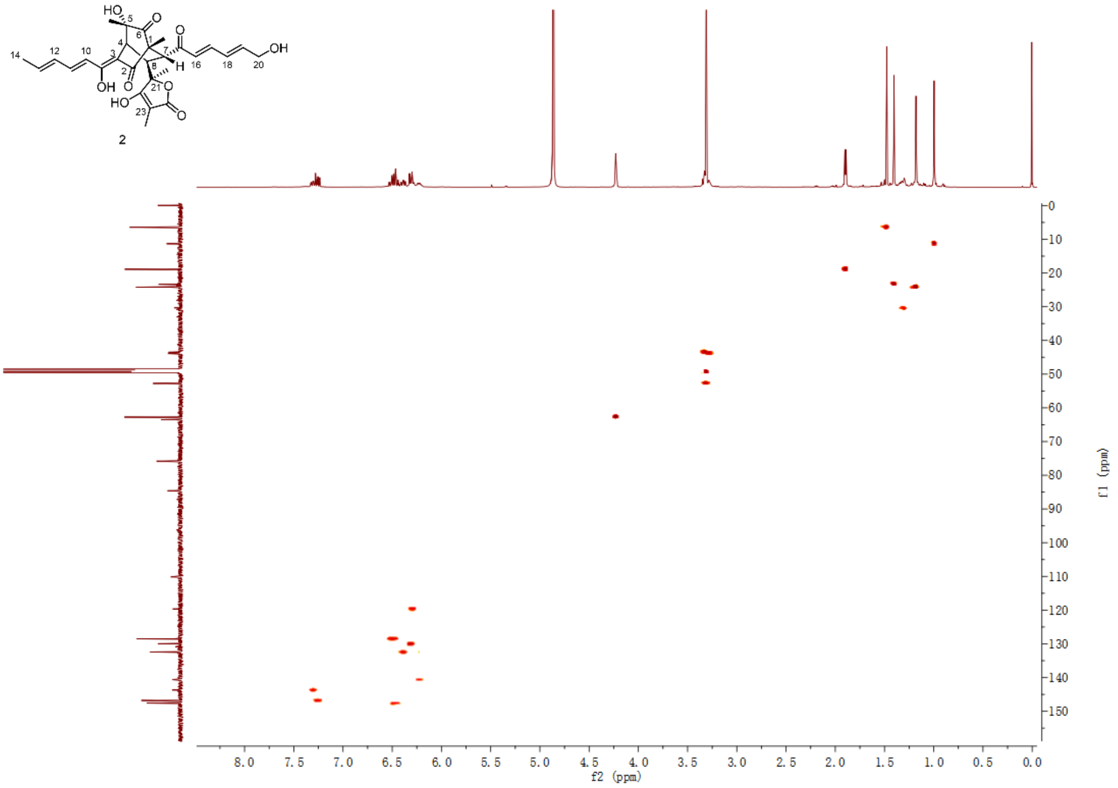

Figure.S11. HSQC spectrum (methanol-d_4, 600 MHz) spectrum of compound **2**

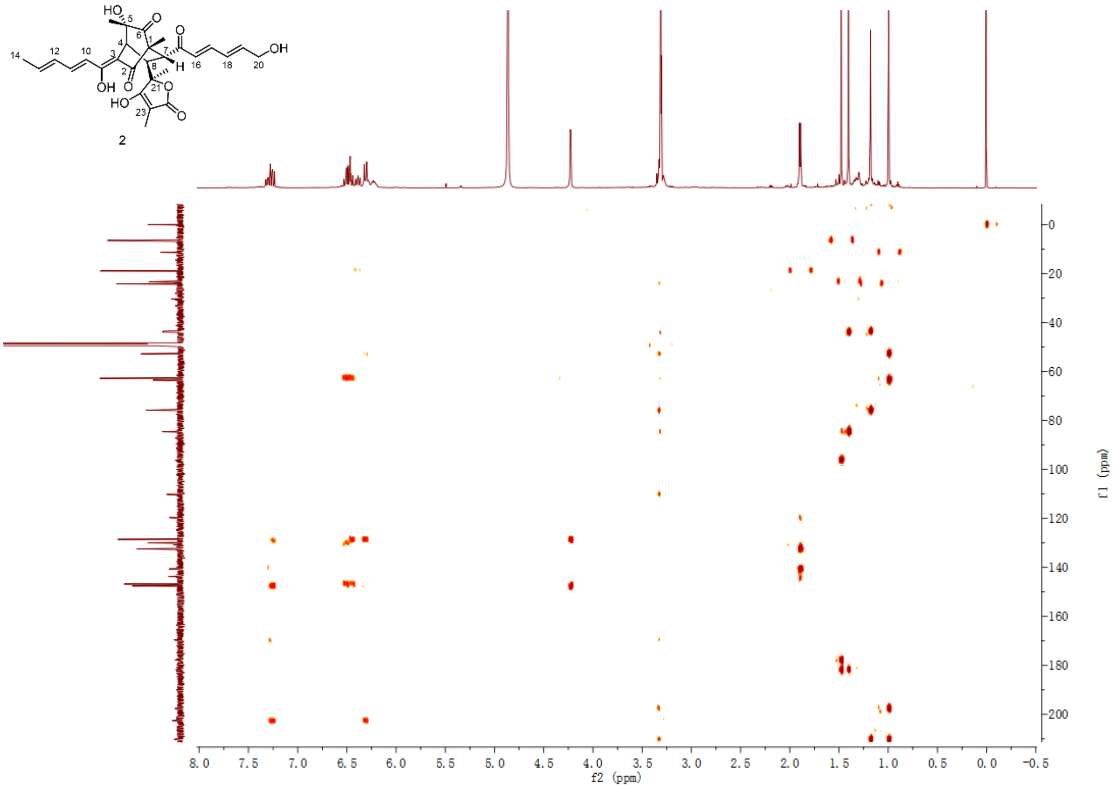

Figure.S12. HMBC spectrum (methanol-d_4, 600 MHz) spectrum of compound **2**

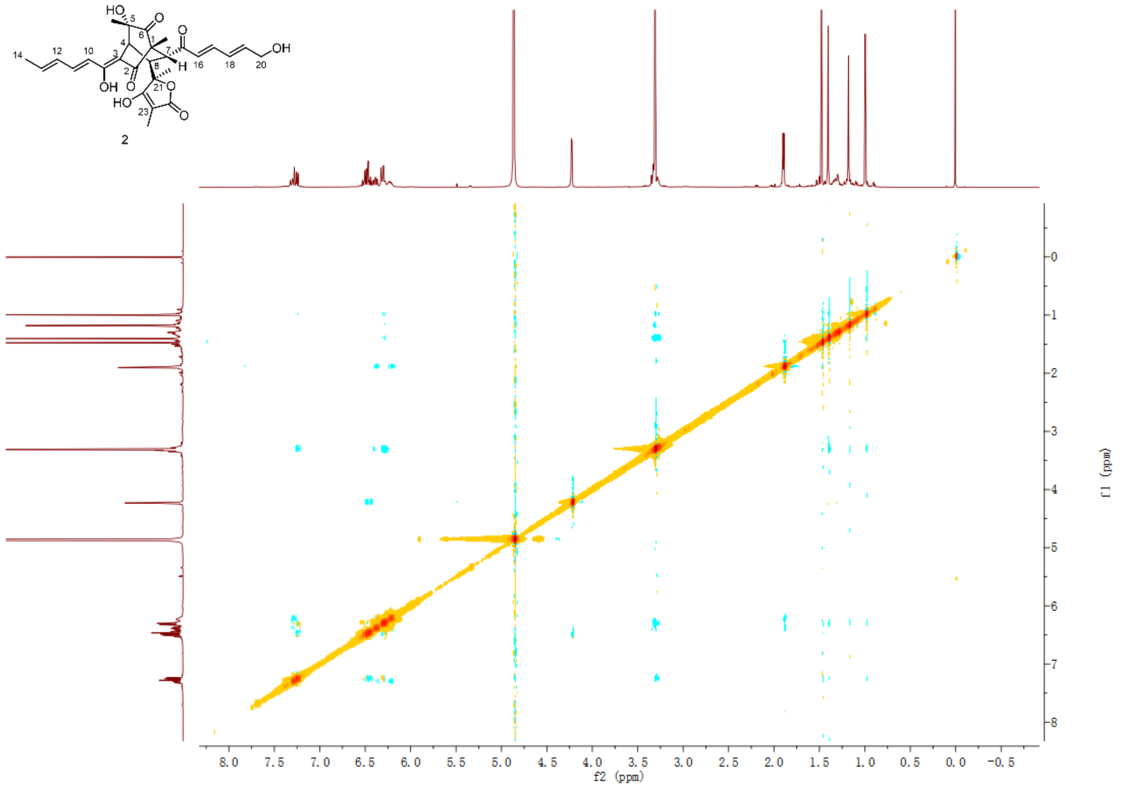

Figure.S13. NOESY spectrum (methanol-d_4, 600 MHz) spectrum of compound **2**

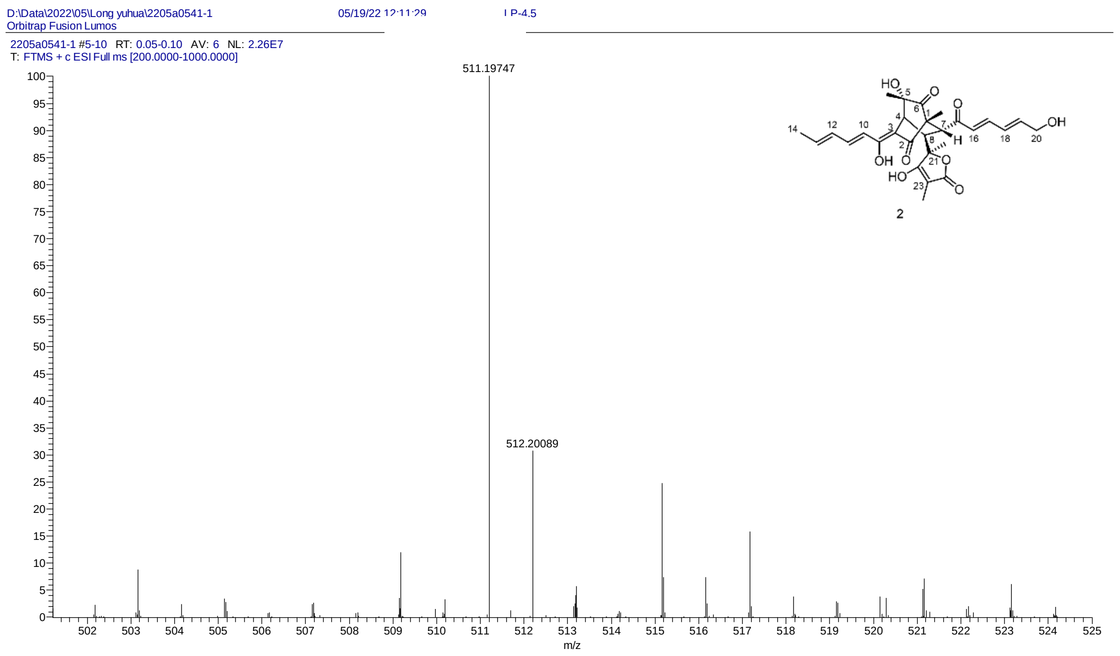

Figure.S14. HR-ESI-MS spectrum of compound **2**

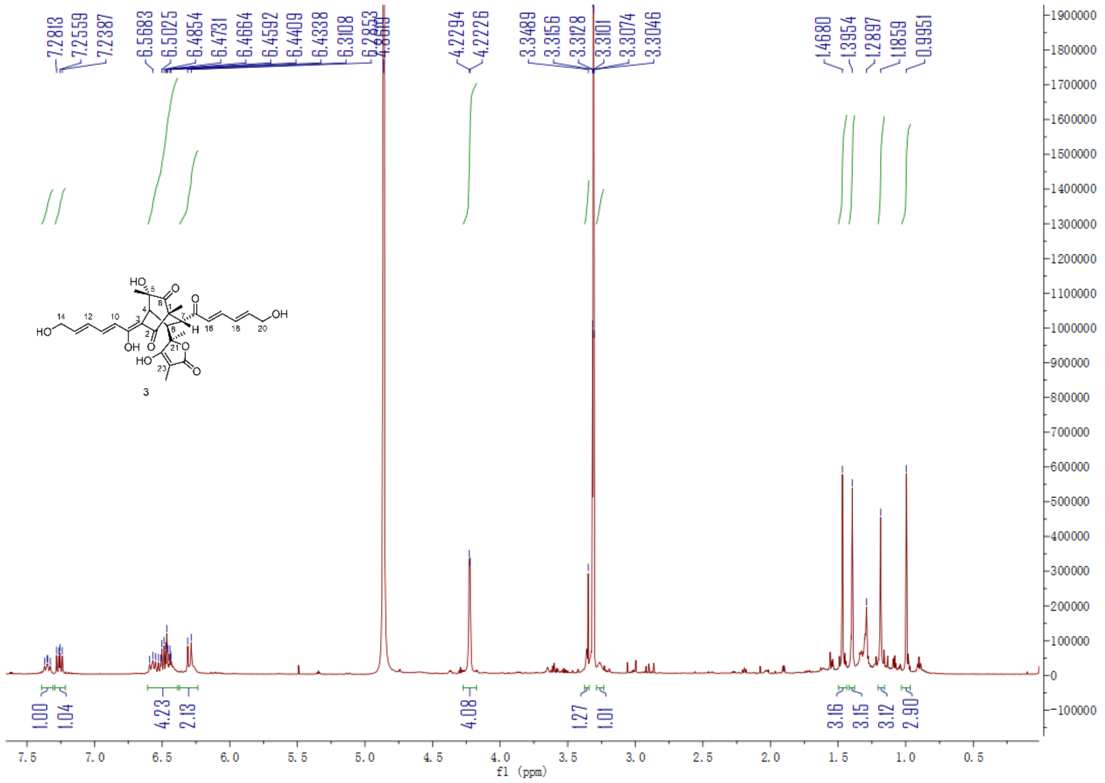

Figure.S15. ^{1}H NMR (methanol-d_4, 600 MHz) spectrum of compound **3**

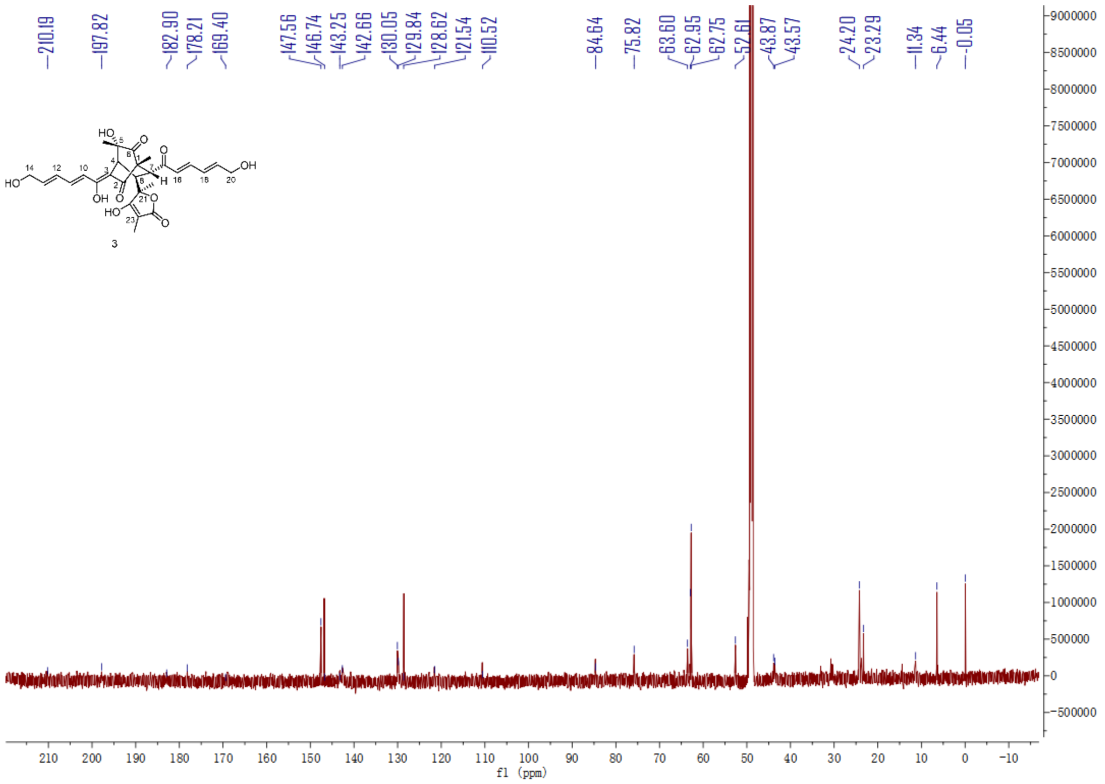

Figure.S16.^{13}C NMR (methanol-d_4, 150 MHz) spectrum of compound **3**

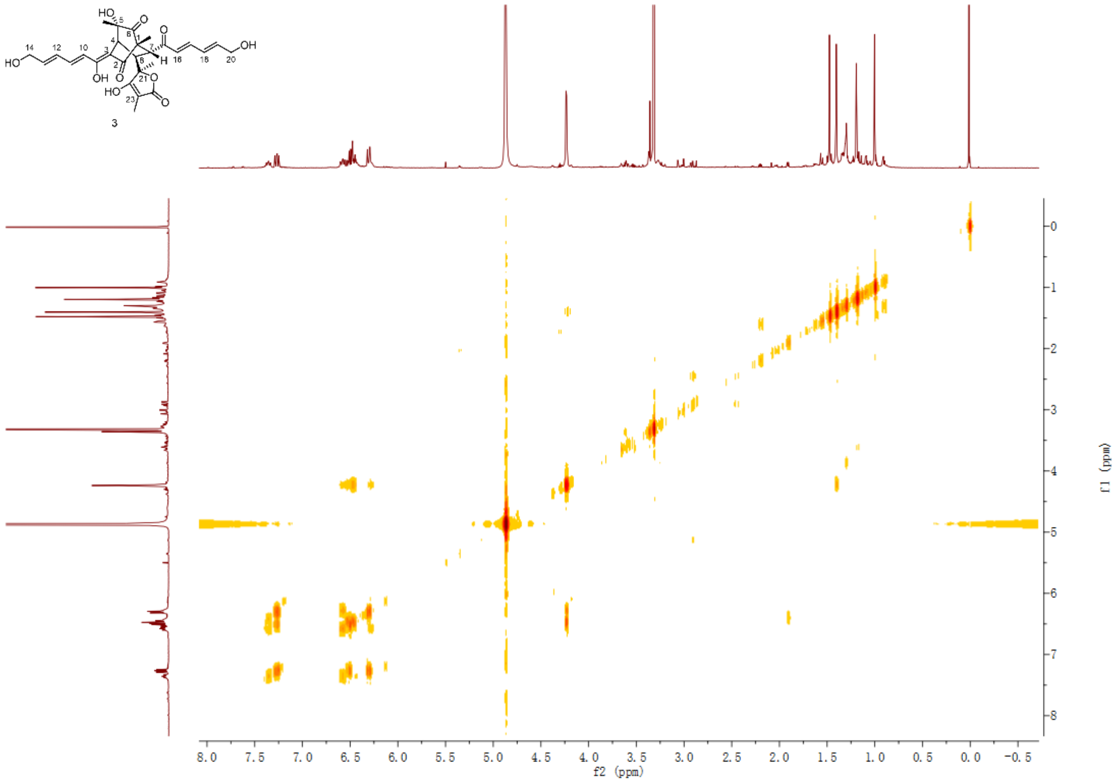

Figure.S17. ^{1}H, ^{1}H- COSY spectrum (methanol-d_4, 600 MHz) spectrum of compound **3**

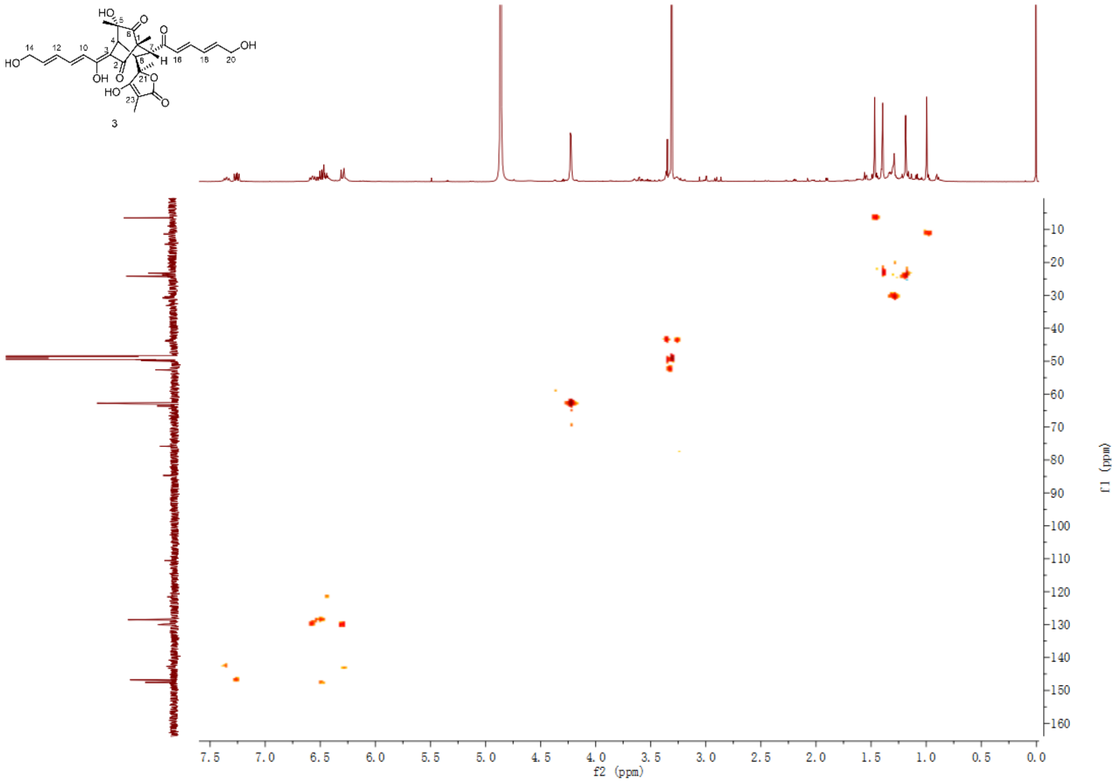

Figure.S18. HSQC spectrum (methanol-d_4, 600 MHz) spectrum of compound **3**

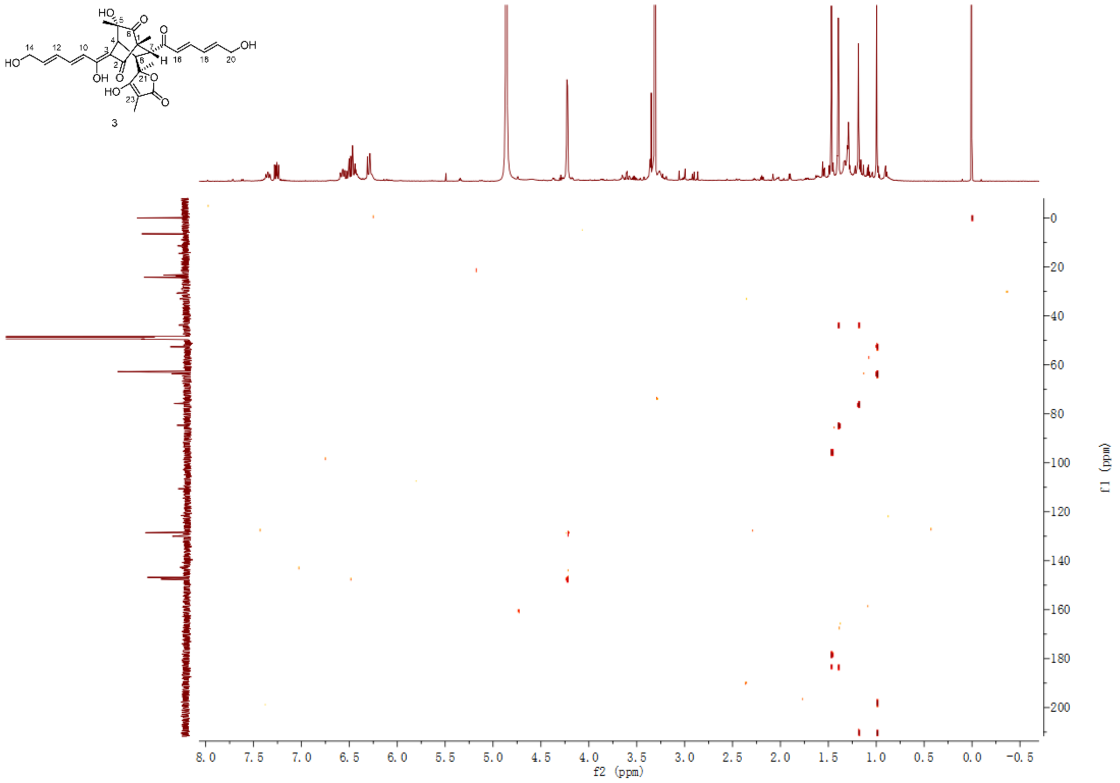

Figure.S19. HMBC spectrum (methanol-d_4, 600 MHz) spectrum of compound **3**

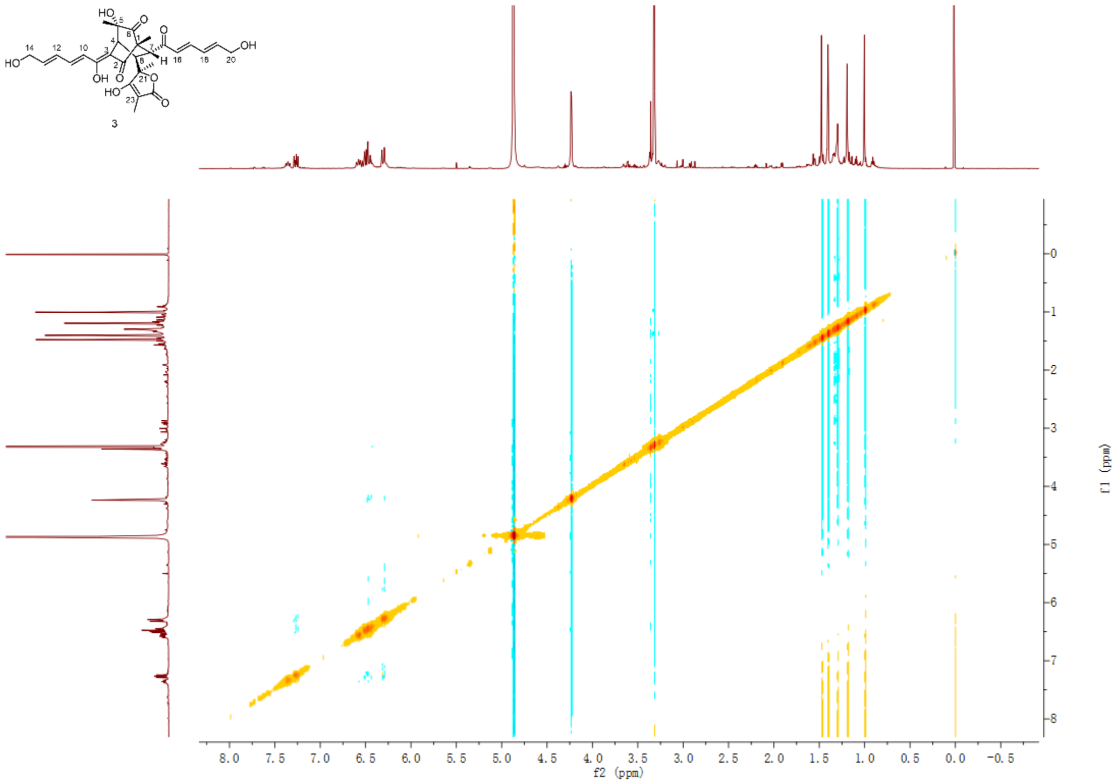

Figure.S20. NOESY spectrum (methanol-d_4, 600 MHz) spectrum of compound **3**

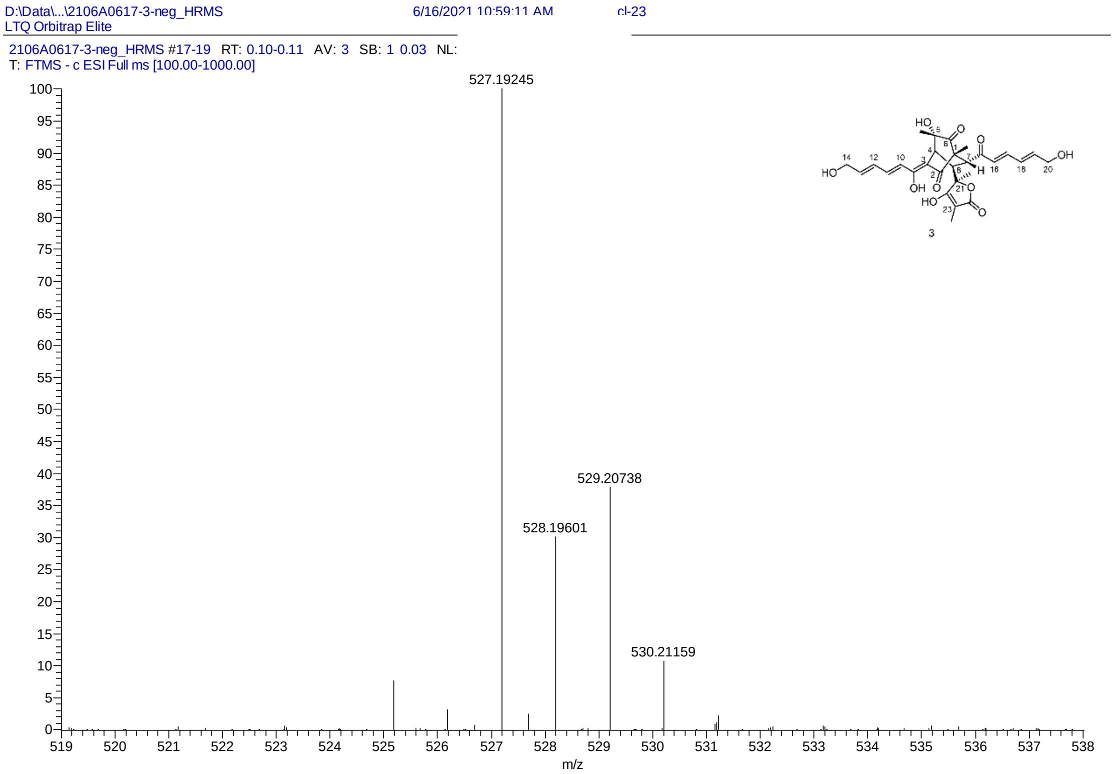

Figure.S21. HR-ESI-MS spectrum of compound **3**

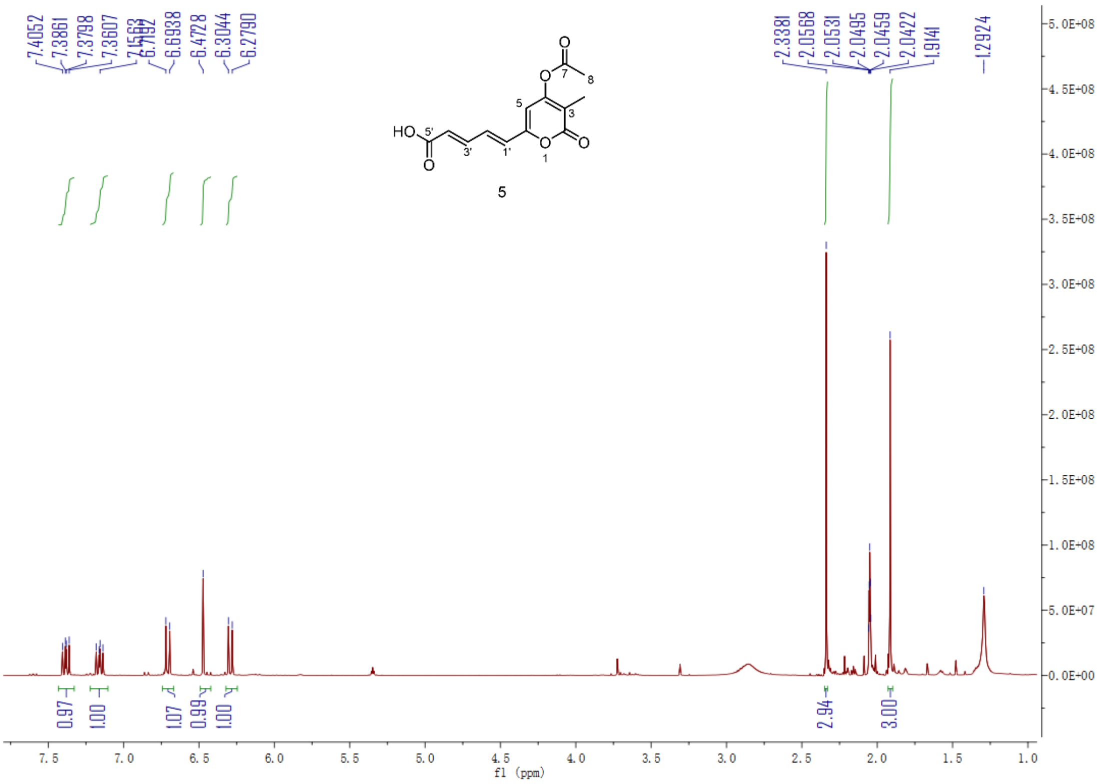

Figure.S22. ^{1}H NMR (acetone-d_6, 600 MHz) spectrum of compound **5.**

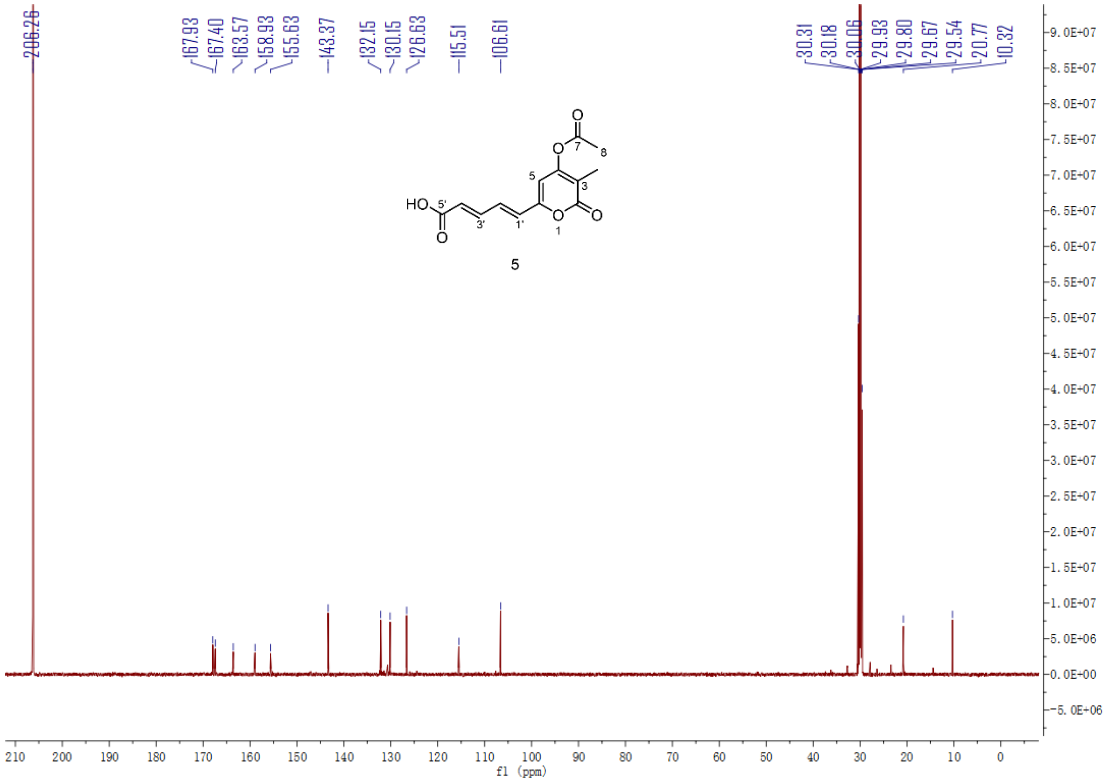

Figure.S23.^{13}C NMR (acetone-d_6, 150 MHz) spectrum of compound **5**

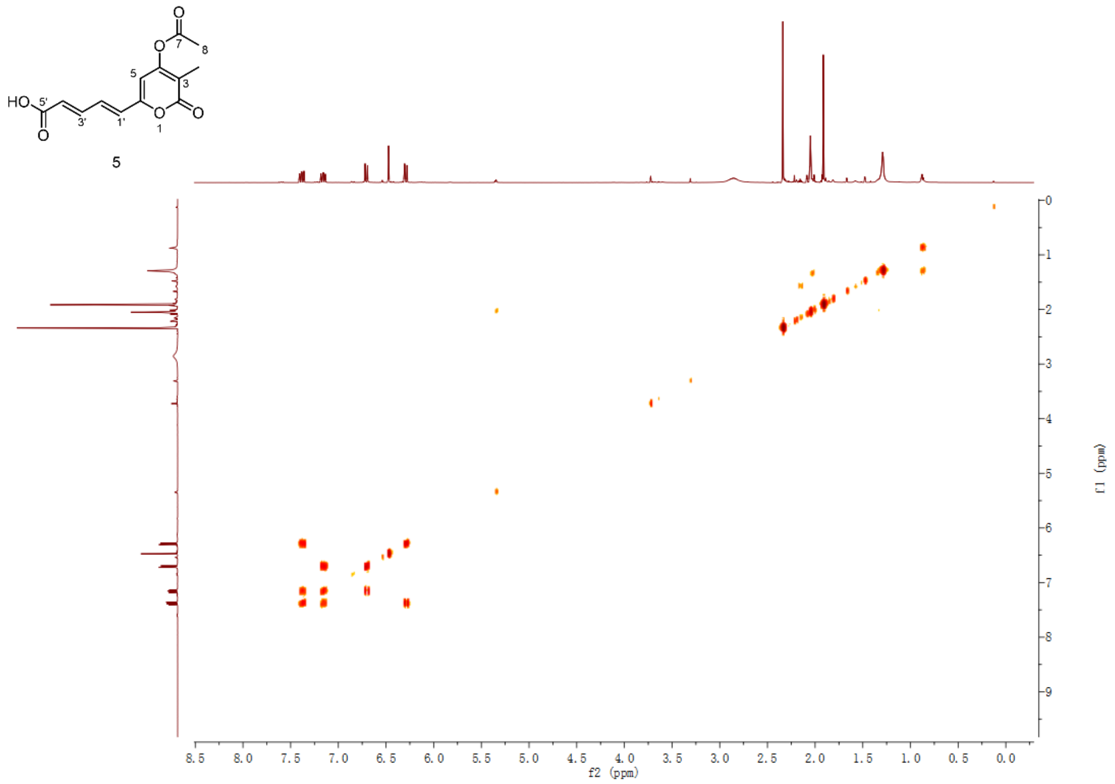

Figure.S24. ^{1}H, ^{1}H- COSY spectrum (acetone-d_6, 600 MHz) spectrum of compound **5**

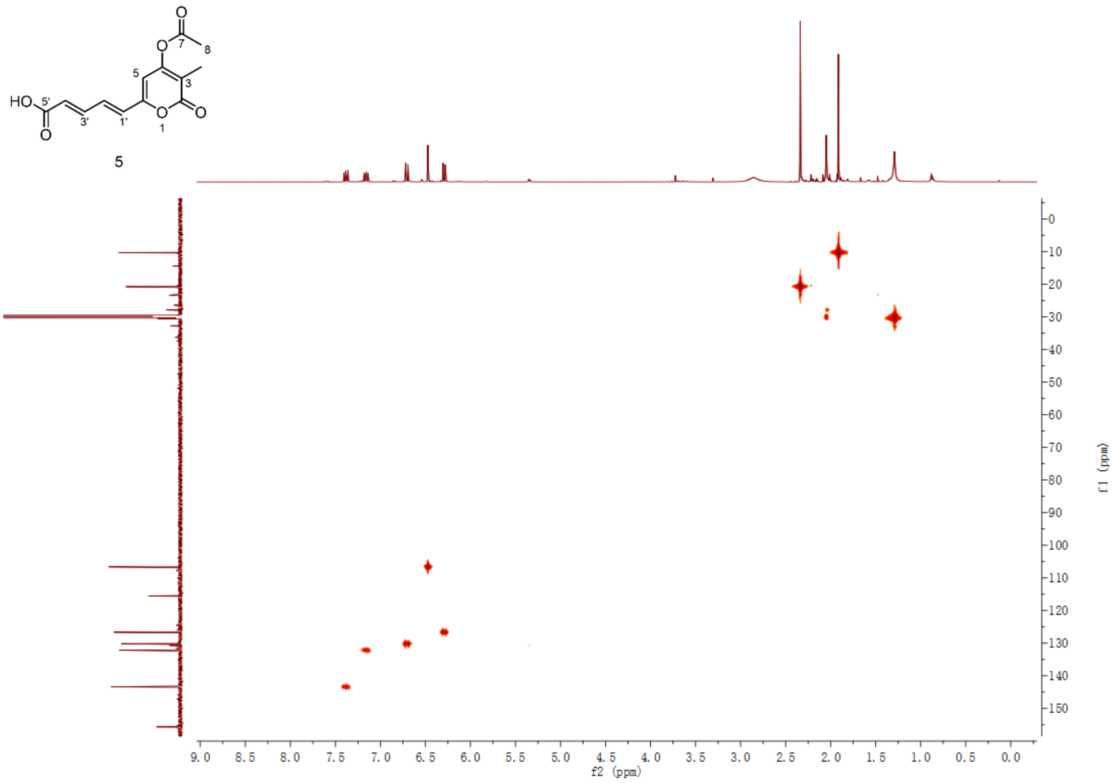

Figure.S25. HSQC spectrum (acetone-d_6, 600 MHz) spectrum of compound **5**

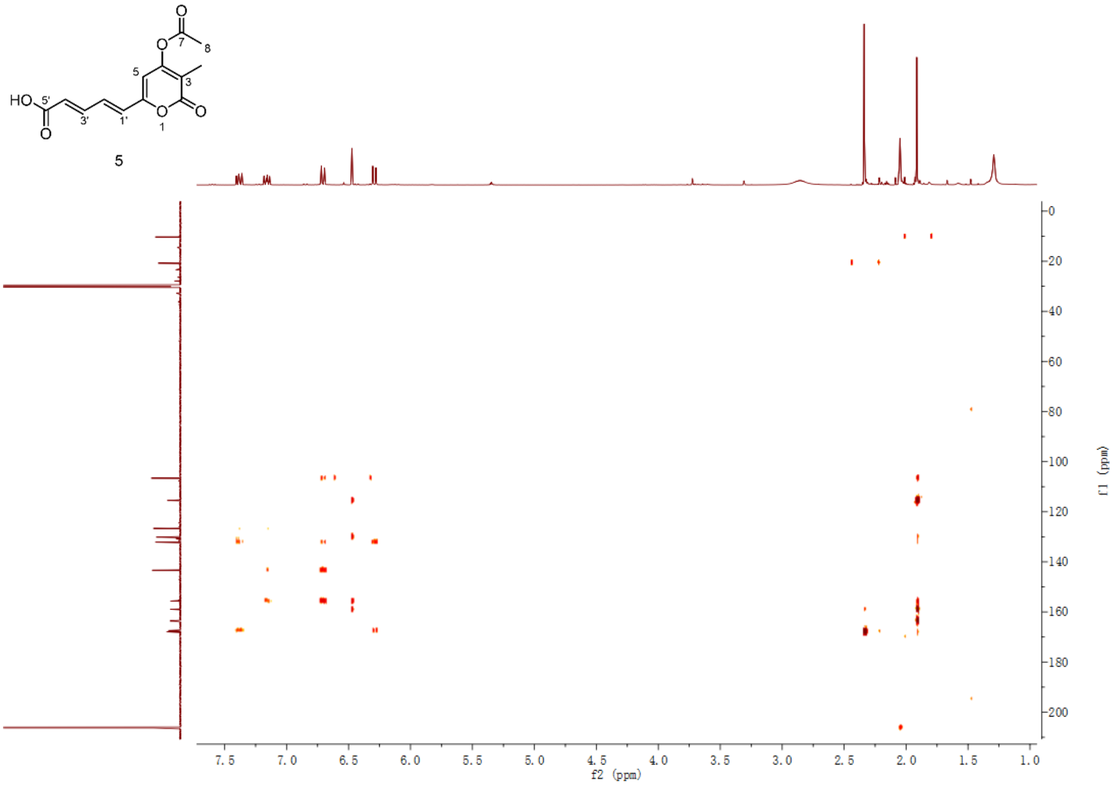

Figure.S26. HMBC spectrum (acetone-d_6, 600 MHz) spectrum of compound **5**

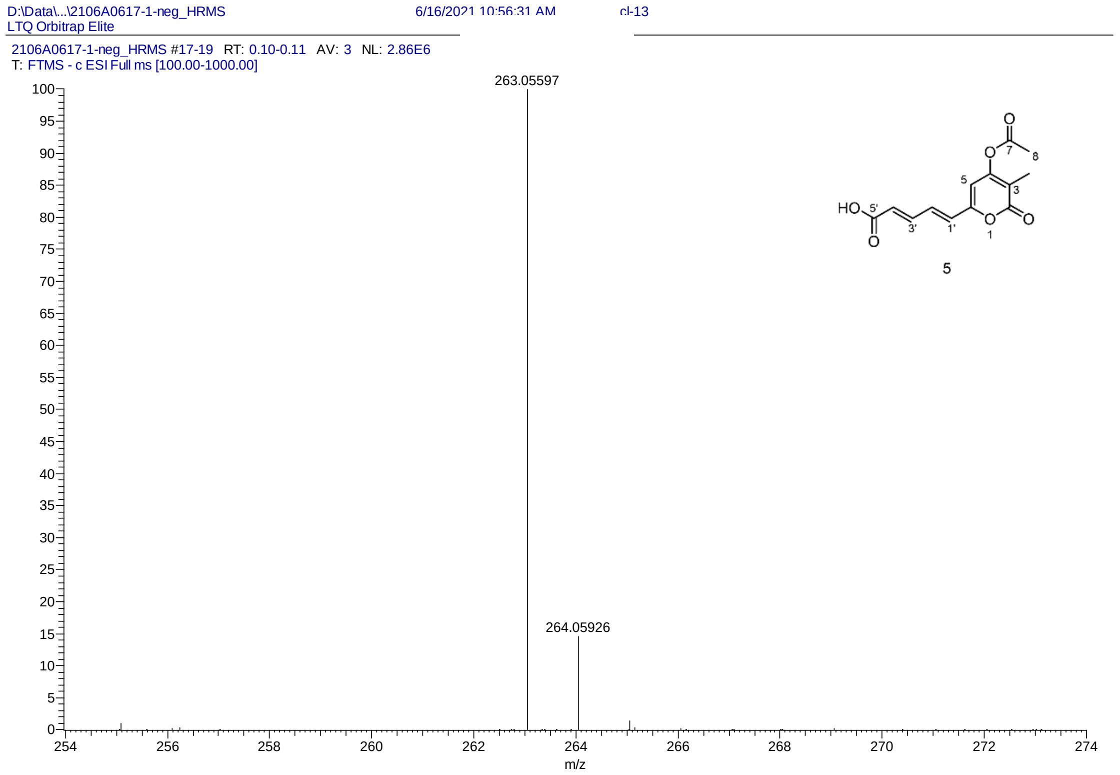

Figure.S27. HR-ESI-MS spectrum of compound **5**

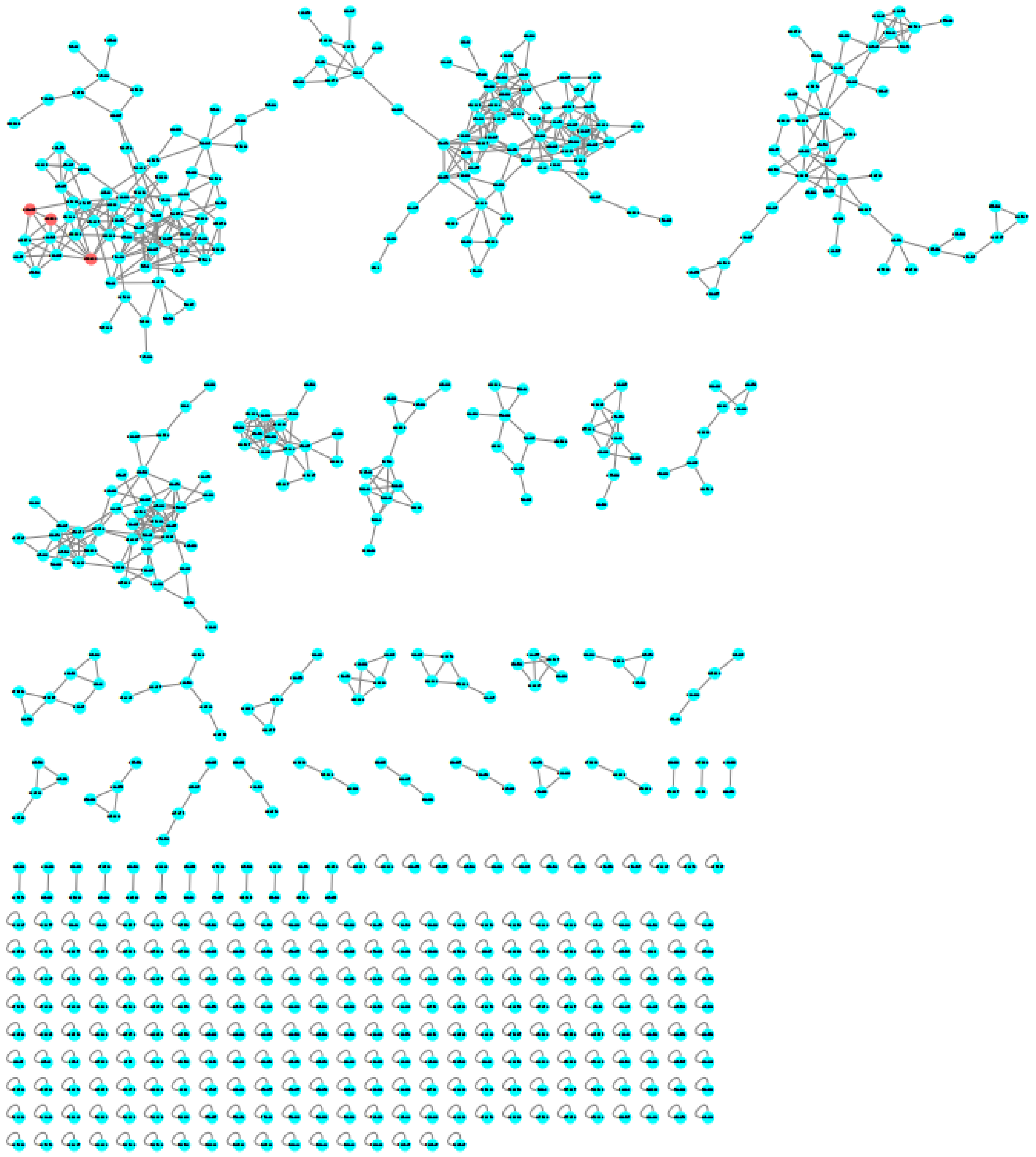

Figure.S28. Molecular networking of the EtOAc extract from strain *Trichoderma reesei SCNU-F0042*.
